# Lactulose with synergists supplementation improving a health of chicks and reducing the environmental burden in poultry industry

**DOI:** 10.5455/javar.2024.k792

**Published:** 2024-06-10

**Authors:** Ivan Fiodorovich Gorlov, Marina Ivanovna Slozhenkina, Daria Aleksandrovna Mosolova, Lyudmila Viktorovna Khoroshevskaya, Zoya Borisovna Komarova, Vladimir Nikolaevich Nikulin, Evgeniya Aleksandrovna Struk, Aleksey Petrovich Khoroshevsky, Elena Yurievna Anisimova

**Affiliations:** 1 Povolzhsky Research Institute of Manufacture and Processing of Meat and Dairy Product, Volgograd, Russian Federation; 2 Universite Paris-Est Creteil Val de Marne Institut d’Administration des Entreprises Gustave Eiffel—Ecole de management, Creteil, France; 3 Orenburg State Agrarian University, Orenburg, Russian Federation

**Keywords:** Excreta noxious gas, gut microflora, lactulose, nutrition, poultry industry

## Abstract

**Objective:**

The study aims to understand the effect of new antibiotic-substituting supplements in feeding chickens of the Hisex Brown cross in industrial conditions.

**Materials and Methods:**

A total of 216 hatched chicks were randomly selected and distributed into Control, Test I, and Test II groups, with 3 replicates of 24 birds in three treatments.

**Results:**

At the end of the experiment, BW of T1/T2 birds was higher by 6.12% (*p *<0.01) and 10.29% (*p <*0.001) than CON. In comparison with the control hens, T1/T2 birds had a higher feed conversion rate and digestibility of nutrients. The blood indicators of T1/T2 hens exceeded those in control. Prebiotic supplementations were positively influenced in the immune indices of birds. IgA, IgG, IgM increased in groups T1/T2. Similar regularity was found in the natural resistance of chicks fed S1/S2. In the caecum, the *Lactobacilli* number was higher than in CON by 17.03% (*p <*0.01) in T1 and by 18.47% (*p <*0.01)—in T2; *Bifidobacteria*—by 17.94 (*p <*0.001) and 19.09% (*p <*0.01), respectively; at the same time, the number of *E. coli* decreased by 21.05% (*p <*0.01) and 24.21% (*p <*0.01). The concentration of emitted excreta noxious gases decreased: ammonia by 22.40%–24.95% (*p <*0.01); hydrogen sulfide by 10.67%–16.00% (*p <*0.01); and mercaptans by 12.90%–17.74% (*p <*0.05).

**Conclusion:**

These findings support the use of lactulose-based supplements as antibiotic alternatives to improve production in poultry farming and to reduce the toxic load on the environment.

## Introduction

The total exclusion of antibiotics from modern industrial poultry farming caused a task to increase the resistance of the body and realize the maximum bioresource potential genetically determined, which remains a serious challenge for Russian poultry enterprises. GI tract-related diseases mainly cause economic losses and severe conditions end up with death [1]. Russia has just started developing the sector of organic livestock and poultry production, so overseas experience is important for our country. Following the trends of organic agriculture, specialists completely or partially reject antibiotics and replace them with bioactive substances of various groups. The scientific community around the world pays great attention to the search for alternatives to antibiotics, which not only positively affect the growth and development of the beneficial microflora in the gastrointestinal (GI) tract and preserve its integrity, but also can increase the protective function of the body and its resistance to pathogenic and conditionally pathogenic microorganisms [2–7]. The positive effects of prebiotic complexes are realized through three main mechanisms, i.e., strengthening the barrier function of the gut due to their interaction with epithelial and immune system cells located in the GI tract, affecting the gut microbiota, and modulating the immune response [8,9].

One of the most effective prebiotic agents is lactulose, a disaccharide that is resistant to cleavage in the upper gut due to the lack of appropriate saccharolytic enzymes, but undergoes anaerobic fermentation by the colonic microbiota, serves as a prebiotic substrate and increases amounts of *Bifidobacteria*, *Lactobacilli,* and bacterial metabolites [10]. Since the amount of beneficial microorganisms (*Bifidobacteria* and *Lactobacilli*) increases, while the number of pathogenic bacteria (*Clostridia*, *Salmonella,* and *E. coli*) decreases, studies on lactulose as a prebiotic are of scientific and practical importance.

However, the laboriousness of the technology and environmental damage from lactulose production have recently remained an unresolved problem; therefore, this prebiotic used in feeding farm animals and poultry was not feasible [11].

Due to the substantiated urgency of the problem of increasing the industry profitability and insufficient information on lactulose-containing supplements used as antibiotic alternatives in industrial poultry farming, a goal was set to study the effect of feed lactulose applied separately or in combination with biologically active synergists (organic acids) on the health of breeding chickens for the reproduction flock of the Hisex Brown cross.

## Materials and Methods

### Ethical approval

The authors confirm that they have followed EU standards for the protection of animals used for scientific purposes. Experiments were conducted in a manner that avoided unnecessary discomfort to the animals by the use of proper management and laboratory techniques. Approval number: EA NIIMMP # 1-2022-01-10.

### Poultry facility and birds

**Figure 1.**
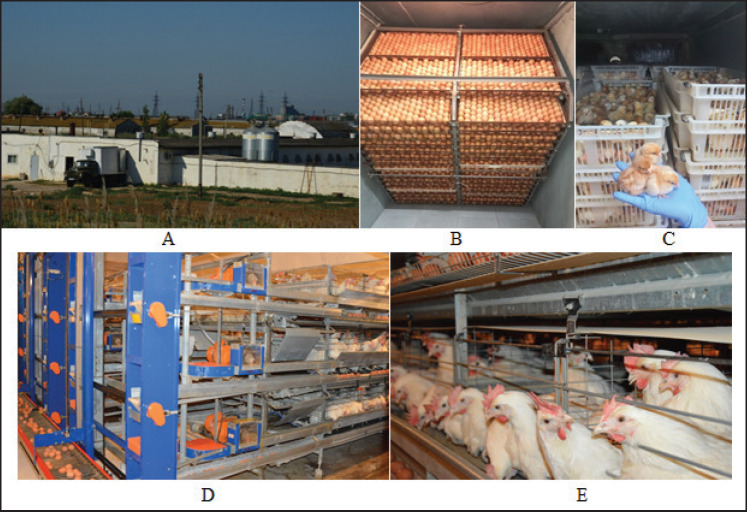
Poultry farm: A is the general view of the enterprise; B is the hatchery section; C is the one-day-old chickens; D is the technological lines; and E is the adult stock.

The experimental studies were conducted on breeding chickens of the parent stock of the Hisex Brown cross in production conditions of the second-order breeding reproducer at the agricultural enterprise “Svetly” (Joint-Stock Company Agrofirma “Vostok”) which is the largest poultry enterprise in the Southern Federal District located in the arid area of Russian Federation (Fig. 1).

A total of 216 hatched chicks were randomly selected and distributed into Control, Test I, and Test II groups, with 3 replicates of 24 birds in three treatments. All the necessary international veterinary and sanitary requirements established for growing chickens were observed.

Chicks were fed a commercial diet ad libitum (Table 1) according to their age; drinking water was freely available.

In the design of our experiment, the replacement young poultry was given a broad-spectrum antibiotic “Eriprim” (S.P.Veterinaria, S.A., Spain) at the rate of 1 kg per 1,000 l of water in the first 5 days after hatching to prevent mycoplasmosis and various coccal infections.

The feed antibiotic Zinc Bacitracin (commercial name “Albac Granular 15%”, Lifecome Biochemistry Co., Ltd., China), compatible with a broad-spectrum antibiotic, was added to the feed in a dose of 0.33 kg per 1 ton of feed, which corresponded to 50 mg of zinc bacitracin per 1 kg of feed, and was included only in control diet during 1–17wks period.

**Table 1.** Composition and nutritional value of poultry diet depending on the age.

Basic diet				
Wheat, barley, corn, soybean meal, sunflower meal, vegetable oil, lysine monochlorohydrate, DL-methionine, L-threonine, monocalcium phosphate, limestone powder, table salt Vitamin and mineral premix for rearing young chickens −1.0% Natugrain TS enzyme				
**Ingredients, %**	**0–5 wks**	**5–10 wks**	**10–17 wks**	
Metabolizable energy, MJ kg^-1^	11.72	10.55		11.63
Crude protein	18.41	14.55		15.25
Crude fat	2.44	2.8		2.38
Linoleic acid	1.18	1.37		1.18
Crude fiber	3.25	5.97		5.07
Lysine	1.0	0.57		0.69
Digestible methionine	0.44	0.26		0.32
Digestible methionine + cysteine	0.83	0.53		0.58
Digestible threonine	0.74	0.49		0.59
Calcium	0.96	0.91		1.45
Phosphorus	0.62	0.58		0.56
Digestible phosphorus	0.42	0.42		0.43
Potassium	0.75	0.65		0.64
Sodium	0.18	0.19		0.18
Сhlorine	0.20	0.19		0.18
NaCl	0.24	0.25		0.22

**Table 2.** The experimental design.

Group	Composition of the diet
CON^1^	Basic diet + feed antibiotic* according to the treatment and prophylactic scheme for growing replacement chicks at the age of 1 to 17 wk + single application of a broad-spectrum antibiotic* for replacement chicks at the age of 2–5 days
T1^2^	Basic diet + S1^4^ 0.5% of the daily ration for 4 to 17 weeks + single application of a broad-spectrum antibiotic** for replacement chicks at the age of 2–5 days
T2^3^	Basic diet + S2^5^ 0.5% of the daily ration for 4 to 17 weeks + single application of a broad-spectrum antibiotic** for replacement chicks at the age of 2–5 days

Note: ^1^ CON is Control group (with a feed antibiotic); ^2^ T1 is Treatment I, Test group I (without a feed antibiotic); ^3^ T2 is Treatment II, Test group II (without a feed antibiotic); ^4^ S1 is Supplement I, prebiotic lactulose derived from milk molasses using an innovative resource-saving technology; ^5^ S2 is Supplement II, a complex based on prebiotic lactulose + biologically active synergists.

* Zinc Bacitracin (“Albac Granular 15%”, Lifecome Biochemistry Co., Ltd., China);

** “Eriprim” (S.P.Veterinaria, S.A., Spain).

The nutritional value of feed for replacement chicks corresponded to the Guidelines for working with the birds of the Hisex Brown cross [12]. The experimental design is shown in Table 2.

The S1 diet contained prebiotic lactulose separately, and the S2 diet contained prebiotic lactulose combined with organic food supplements as biologically active synergists, i.e., aminoacetic acid—glycine (E640, Mixem, Russia), ascorbic acid (E300, Mixem, Russia), and malic acid (E296, Mixem, Russia).

### Parameters under study

Bird weighing with data registration was carried out weekly using a special portable scale (FlexScale, Big Dutchman Inc., USA) according to the methodologies of the farm and recommended by the manufacturer of the cross.

The digestibility and utilization of nutrients in birds were evaluated by the physiological experiment [13]. The bioconversion of feed nutrients was studied in accordance with the guidelines developed by the Federal Scientific Center “All-Russian Research and Technological Institute of Poultry Farming” Russian Academy of Sciences. The nutritional value of the feed was monitored using an automatic infrared analyzer SpectraStar 2,000 (Unity Scientific, USA).

To study the hematological parameters and immune status of the body, 9 chickens at the age of 4 wk and the age of 17 wk (before being transferred to a mature productive flock) were selected from each group. Blood was sampled from the subdermal cubital vein on the inner surface of the wing before feeding in the morning. Morphological analysis was conducted by automatic analyzer URIT-3020 Vet and biochemical analysis—by semi-automatic analyzer URIT-800 Vet (Urit Medical Electronic Co., Ltd., China).

The natural resistance indicators of the chicken body—BA, LA, and PA—were determined by the procedures proposed by Deryabin and Polyakov [14], Fogelson et al. [15], and Shirshev et al. [16], respectively.

IgA, IgG, and IgM concentrations were determined by enzyme immunoassay using chicken ELISA-Kits (Bethyl Laboratories^®;^, Inc., USA) in accordance with the manufacturer’s protocol (Cat. No. E33-103, E33-104, and E33-102, respectively).

For gut microbiota analysis from chickens at 17 wk of age, 8 samples of GI contents were taken from caecums in the intestine and then the composition was determined by Terminal Restriction Fragment Length Polymorphism [17].

The amount of excreted litter was determined by weighing it on a portable electronic scale CAS SW-10W (CAS Co., Ltd., Korea) with respect to the age of the birds.

The content of noxious gases emitted by manure litter (chicken litter of laying hens at the age of 17 wk) was found by the method described by Tang et al. [18]. To measure the concentration of excreta noxious gases, a GV-100S hand-held pumping sampler (Gastec Corp., Japan) was used with appropriate indicator tubes (Ammonia No. 3L 30–78 ppm, Hydrogen sulfide No. 4LT 2–20 ppm and total mercaptan (R-SH) No. 70L 4–8 ppm).

### Statistical data processing

All digital data were processed using the statistical software Statistika 12.0 (Statsoft Inc., USA) and Student’s *t*-test to compare the mean values of experimental groups with the control group (Johnson and Bhattacharyya, 2010). Differences of *p* < 0.05 were considered significant.

## Results and Discussion

### Growth performance and feed intake indices

**Figure 2.**
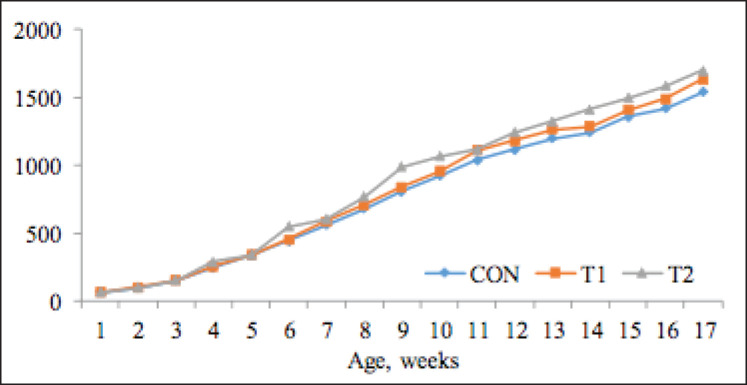
BW dynamics, gm (*n =* 24).

The livability of chickens in all groups was 100% throughout the experiment. In comparison with control birds, T1/T2 chickens have a higher BW by the end of the experiment. Figure 2 shows the BW of chickens (1 to 4 wk of age) to be at the same level before supplementation. In the age period of 4 to 17 wk (until the transfer to the adult flock), BW significantly exceeded the control; the difference was in favor of T1 and T2 birds at the age of 17 wk and made 6.12% (*p<*0.01) and 10.29% (*p<*0.001), respectively.

A slight upward trend in the body weight (BW) when feeding lactulose to broiler chickens was found by Calik and Ergün [7] and when feeding turkeys by Santana et al. [19]. Perhaps this could be explained by the insufficient duration of the experiment (42 days) or the low dose of the prebiotic administrated. In contrast, Cho and Kim [4] and Hossain et al. [20] observed in weaned piglets fed supplements a significant gain in weight compared to the control without treatment.

Based on the development and BW gains of the experimental young poultry data, it is possible to state that feeding of poultry for breeding with standard feed portions, exactly dosed according to the cross manufacturer’s feeding plan, in combination with the introduction of the studied feed additives (T1 and T2) based on the lactulose prebiotic and other nutritional components into the diet increased the digestive enzymes activity of the small intestine in the body of experienced poultry relative to enzymes activity of the young birds of control group intestine, which leads to more complete decomposition of hardly hydrolyzable feed components to a digestible form and more complete digestion of the diet used nutrients. The identified trend can be observed when analyzing the doses of factual feed intake and conversion for each group during the experiment (Table 3).

The nutrient digestibility improvement may be due to the lactulose capacity revealed in previous studies to increase the absorption rate and optimize the proportion of energy/protein in the body by increasing the length of the intestine and changing its morphological parameters, i.e., the width and area of its surface due to an increase the height of the villi [5,7,19].

**Figure 3.**
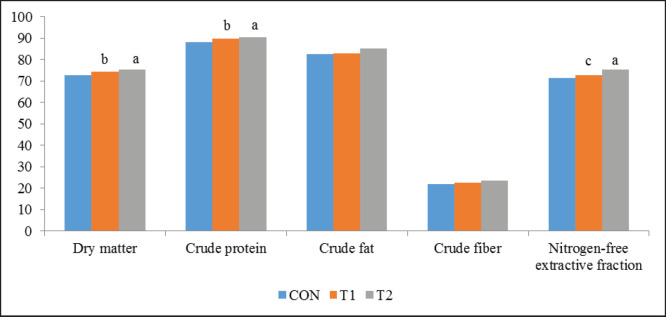
Digestibility of feed nutrients, %.

The digestibility coefficients of T1 and T2 hens were higher than those of Control hens, i.e., in terms of the dry matter (DM) by 2.34% (*p <*0.01) and 3.43% (*p <*0.001); Crude Protein by 1.70% (*p <*0.01) and 2.49% (*p <*0.001); nitrogen-free extractive fraction by 1.68% (*p <*0.05) and 5.31% (*p< *0.001); crude fat by 0.48% (ns) and 3.27% (*p<*0.001); and Crude Fiber by 3.20% (*p <*0.001) and 7.76% (*p <*0.001), respectively (Fig. 3).

Differences in the crude feed protein digestibility under the studied feed supplements influence affected nitrogen metabolism in birds (Table 4). This means that lactulose improves the well-being of the birds and reduces digestive problems and weight loss caused by different negative factors [5,7].

**Table 3.** Feed intake and feed conversion rate of groups over the 17–week experimental period (mean ± S.E.M.).

Indicator	Group		
	CON (*n =* 24)	T1 (*n =* 24)	T2 (*n =* 24)
Feed intake per chicken (0–5 wks), g	875.2 ± 5.9	870.1 ± 4.2	872.4 ± 6.5
Feed conversion per chicken (0–5 wks)	2.30 ± 0.02	2.28 ± 0.02	2.27 ± 0.01
Feed intake per chicken (6–10 wks), g	1799.4 ± 10.1	1788.3 ± 8.8	1790.1 ± 11.2
Feed conversion per chicken (6–10 wks)	2.04 ± 0.01	2.01 ± 0.01^c^	1.95 ± 0.02^a^
Feed intake per chicken (11–17 wks), g	3341.0 ± 9.5	3350.3 ± 6.8	3346.0 ± 7.5
Feed conversion per chicken (11–17 wks)	2.23 ± 0.01	2.17 ± 0.02^b^	2.15 ± 0.02^a^
Total feed intake per chicken over 17 wks, g	6015.6 ± 10.6	6008.7 ± 8.4	6008.5 ± 9.1
Feed conversion per chicken over 17 wks	4.21 ± 0.02	4.15 ± 0.01^b^	4.09 ± 0.03^b^

Note: a = *p* < 0.001; b = *p* < 0.01; and c = *p* < 0.05 compared with data on control group.

**Table 4.** Utilization of nitrogen by bird, g (mean ± S.E.M.).

Indicator	Group		
	CON (*n =* 24)	T1 (*n =* 24)	T2 (*n =* 24)
Consumed nitrogen	3.21 ± 0.01	3.21 ± 0.01	3.21 ± 0.01
Excreted with feces	1.22 ± 0.12	1.10 ± 0.06	1.09 ± 0.05
Assimilable nitrogen	1.99 ± 0.07	2.11 ± 0.04	2.12 ± 0.03
Absorbed from consumed, %	61.99 ± 0.44	65.73 ± 0.53^a^	66.04 ± 0.42^a^

Note: a = *p* < 0.001; b = *p* < 0.01; and c = *p* < 0.05 compared with data on control group.

**Table 5.** Morphological and biochemical blood profile (17 wks of age, mean ± S.E.M.).

Parameter	Group		
	CON (*n =* 24)	T1 (*n =* 24)	T2 (*n =* 24)
Erythrocytes, 10^12^/l	2.79 ± 0.11	3.17 ± 0.09^b^	3.21 ± 0.13^c^
Leukocytes, 10^9^/l	39.82 ± 0.58	37.21 ± 0.44^a^	36.95 ± 0.38^a^
Hemoglobin, g/l	104.2 ± 1.44	110.1 ± 1.57^b^	111.9 ± 1.51^a^
Hematocrit, %	39.11 ± 0.79	41.75 ± 0.85^c^	41.93 ± 0.91^c^
Basophils, %	2.82 ± 0.12	2.71 ± 0.09^ns^	2.94 ± 0,15^ns^
Eosinophils, %	7.05 ± 0.19	7.10 ± 0.18^ns^	6.82 ± 0.21^ns^
Pseudoeosinophils:			
stab, %	0.22 ± 0.07	0.31 ± 0.08^ns^	0.20 ± 0.05^ns^
segmented, %	27.19 ± 0.41	25.93 ± 0.34^c^	26.03 ± 0.27^c^
Lymphocytes, %	57.71 ± 0.29	58.90 ± 0.35^c^	59.12 ± 0.51^c^
Monocytes, %	5.01 ± 0.21	5.05 ± 0.26^ns^	4.89 ± 0.31^ns^
Total protein, g/l	41.24 ± 0.61	43.52 ± 0.82^c^	43.96 ± 0.75^b^
Albumins, g/l	18.85 ± 0.41	20.74 ± 0.58^b^	20.99 ± 0.59^b^
Globulins, g/l	22.39 ± 0.42	22.78 ± 0.48^ns^	22.97 ± 0.54^ns^
Alkaline phosphatase, U/l	165.21 ± 6.34	141.95 ± 4.51^b^	140.37 ± 5.28^b^
AST^1^, U/l	276.82 ± 8.94	239.36 ± 7.45^b^	235.59 ± 7.17^a^
ALT^2^, U/l	6.52 ± 0.31	5.46 ± 0.27^c^	5.31 ± 0.34^c^
Glucose, mmol/l	5.64 ± 0.19	6.38 ± 0.24^b^	6.29 ± 0.22^c^
Urea, mmol/l	2.95 ± 0.11	3.37 ± 0.09^b^	3.31 ± 0.08^c^
Cholesterol, mmol/l	3.89 ± 0.22	3.25 ± 0.17^c^	3.11 ± 0.19^b^

Note: a = *p* < 0.001; b = *p* < 0.01; and c = *p* < 0.05 compared with data on Control group.

^1^ Aspartate transaminase.

^2^ Alanine transaminase.

### The comparative hematology and biochemical analysis of birds

Chicks at the age of 4 wk had hematological parameters without significant difference. However, at the end of the experiment (chicken aged 17 wk), it was found that erythrocytes in the blood of T1 and T2 hens exceeded those of Control hens by 13.62% (*p <*0.01) and 15.05% (*p <*0.01); hemoglobin content by 5.66% (*p <*0.01) and 7.39% (*p <*0.01); and hematocrit by 2.64% (*p <*0.05) and 2.82% (*p <*0.05), respectively (Table 5). Fast metabolism indicates a high rate of redox reactions based on red blood cells and the hemoglobin they contain.

Among other indicators, leukocytes characterize the immune system of animals and birds. The leukocyte number within the physiological norm indicates a normal physiological state of the body and a fairly strong immunity. In our experiment, the contents of leukocytes in Test groups T1 and T2 compared to the Control group decreased by 6.55% (*p <*0.01) and 7.21% (*p <*0.001), respectively, which indicated a decrease in inflammatory processes in T1 and T2 chickens. At the same time, the leukocyte contents in both the Test and Control groups were within the physiological norm. We should note an increase in lymphocytes in T1 and T2 hens than in CON by 1.26% (*p <*0.05) and 1.51% (*p <*0.05), respectively, with a simultaneous decrease in segmented neutrophils by 1.22% (*p <*0.05) and 1.06% (*p <* 0.05), which convincingly proved high efficiency of the studied preparations as alternatives to feed antibiotics.

The total serum protein of T1 and T2 chickens was 5.53% (*p <*0.05) and 6.60% (*p <*0.05) higher than in CON. The level of albumin fraction in the composition of total protein in T1 and T2 hens exceeded that in CON by 10.03% (*p <*0.05) and 11.35% (*p<*0.01), respectively. The upward trend in the amount of globulin fractions in the blood serum indicated that inflammatory processes were stopped in the chicken’s body and immunity increased; however, the identified differences were not statistically significant. A significant increase in the urea content by 14.24% (*p <*0.01) in T1 hens and by 12.20% (*p <*0.05) in T2 hens compared to CON indicated a more intensive protein metabolism. Moreover, the decrease in the alkaline phosphatase value by 14.08% (*p <*0.01) in T1 chickens and by 15.04% (*p <*0.01) in T2 chickens with respect to the control confirmed the absence of inflammatory processes.

However, Cho and Kim [4] found no significant differences in these indicators on weaned piglets. In contrast, Hossain et al. [20] observed a significant increase in the iron-binding capacity of serum caused by an increase in hemoglobin in weaned piglets fed a diet with lactulose in combination with δ-Aminolevulinic acid and an increase in red blood cells compared to piglets fed with a control diet. The differing results may be influenced by different dietary compositions, the experimental duration, and animal species.

### Immunity indicators evaluation

Table 6 shows that our research established significant differences between the values of natural resistance indicators and confirmed higher efficiency of the adaptive-protective processes in the body. The blood serum values of T1 and T2 chickens were revealed to be significantly superior over the control value by 1.17% (*p <*0.05) and 1.68% (*p <*0.01) in terms of the bactericidal activity and by 2.62% and 2.96% in terms of the lysozyme activity. The index of phagocytic activity was also higher by 7.63% (*p <*0.05) in T1 hens and by 7.77% (*p <*0.01) in T2 hens.

Immunoglobulins are produced by lymphocytes in response to the penetration of foreign harmful substances into a living organism [21], namely, IgM is produced in the primary immune response of B-lymphocytes to a foreign antigen and IgG in the secondary immune response and antitoxic immunity. The IgA production occurs in response to local antigen exposure. Their function is to protect the mucous membranes of the respiratory passages and urogenital and GI tracts from infection.

As Table 7 shows, the contents of all fractions of gamma globulins significantly increased in T1 and T2 groups in comparison with the control and stayed within the physiological norm, i.e., IgA increased by 11.93% (*p *<0.05) and 17.70% (*p *<0.01); IgG by 20.06% (*p <0*.05) and 26.27% (*p *<0.01); and IgM by 11.85% (*p *<0.05) and 17.04% (*p *<0.01), respectively.

Increased concentrations of serum IgG and IgM indicated that the experimental chickens’ humoral immune statuses were better and immunity was stronger, which was consistent with Zhu et al. [22], Amevor et al. [23], and Dilawar et al. [24]. Mannanoligosaccharides and xylooligosaccharides are known to be able to increase local mucosal IgA secretion and humoral and cell-mediated immune responses [21]. An increase in the IgA content in the birds’ blood serum in Test groups compared with the Control group might be due to an increase in beneficial microflora and a decrease in pathogenic and opportunistic microflora, as well as indicate a positive effect of the lactulose-containing diet on the gut immunity. Thus, the studied feed supplements may provide stimulatory effects on the immune system and help strengthen nonspecific immunity [25].

**Table 6.** Indicators of nonspecific resistance of the chicken body (mean ± S.E.M.).

Indicator	Group		
	CON (*n =* 24)	T1 (*n =* 24)	T2 (*n =* 24)
Bactericidal activity, %	50.96 ± 0.27	52.13 ± 0.38^c^	52.64 ± 0.47^b^
Lysozyme activity, %	14.75	17.37	17.71
Phagocytic activity, %	54.58 ± 1.61	62.21 ± 2.13^b^	62.35 ± 1.89^b^

Note: a = *p* < 0.001; b = *p* < 0.01; and c = *p* < 0.05 compared with data on control group.

**Table 7.** Indicators of the immune statuses of birds, mg/ml of serum (mean ± S.E.M.).

Indicator	Group		
	CON (*n =* 24)	T1 (*n =* 24)	T2 (*n =* 24)
IgA	2.43 ± 0.08	2.72 ± 0.11^c^	2.86 ± 0.14^b^
IgG	3.54 ± 0.18	4.25 ± 0.21^c^	4.47 ± 0.23^b^
IgM	1.35 ± 0.04	1.51 ± 0.05^c^	1.58 ± 0.05^a^

Note: a = *p* < 0.001; b = *p* < 0.01; and c = *p* < 0.05 compared with data on control group.

**Table 8.** Composition of chicken gut microbiota, colony-forming unit /gm (mean ± S.E.M.).

Indicator	Group		
	CON (*n =* 8)	T1 (*n =* 8)	T2 (*n* = 8)
*Bifidobacteria*	10.53 ± 0.32	12.42 ± 0.29^a^	12.54 ± 0.41^b^
*Lactobacilli*	12.45 ± 0.34	14.57 ± 0.55^b^	14.75 ± 0.48^b^
*E. coli*	7.27 ± 0.29	5.74 ± 0.35^b^	5.51 ± 0.22^a^

Note: a = *p* < 0.001; b = *p* < 0.01; and c = *p* < 0.05 compared with data on control group.

**Figure 4.**
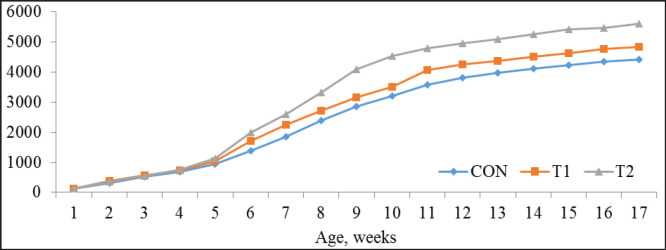
Litter output per day, gm (*n =* 24).

### Gut microbiota analysis

At the age of 17 wk, the gut microbiocenosis of chicks was analyzed (Table 8). The amount of *Lactobacilli* was higher by 17.03% (*p <*0.01) in T1 than in the Control group. Similarly, *Lactobacilli *in T2 exceeded the Control by 18.47% (*p <*0.01). The amount of *Bifidobacteria* in T1 and T2 hens was significantly higher than in CON by 17.94 (*p <*0.001) and 19.09% (*p <*0.01), respectively. In birds fed lactulose-containing supplements decreased the amount of *E. coli*: in T1 and T2 groups than in CON by 21.05% (*p <*0.01) and 24.21% (*p <*0.001), respectively.

The evaluation results of the relationship between the feeding lactulose and the gut microbiome are controversial. Guerra-Ordaz et al. [26] established the effect of a prebiotic on *Lactobacilli* and *Bifidobacteria* in the intestines of piglets. Kamphues et al. [27], however, found no significant effect on the lactic acid bacteria. Cho and Kim [10] reported that *Lactobacilli* increased and *E. coli* decreased in broiler feces of chickens fed with lactulose-containing diets. Maintaining homeostasis in the gut environment is of decisive importance for digestion and absorption of nutrients.

### Amount of litter and noxious gas emission values

We studied the effect of the feed supplements on the amount of litter (Fig. 4) and the concentration of emitted excreta noxious gases—ammonia, hydrogen sulfide, and mercaptans (Table 9).

The gasometric analysis established that amount of emitted excreta noxious gases in control group vs T1/T2 was higher, i.e., ammonia by 22.40% (*p <*0.01) and 24.95% (*p <*0.001); hydrogen sulfide by 10.67% (*p *<0.01) and 16.00% (*p *<0.001); and mercaptans by 12.90% (*p <*0.05) and 17.74% (*p <*0.01), respectively.

Prebiotic preparations used in feeding promote the growth of colonies that ferment carbohydrates, such as *Bifidobacteria* and *Lactobacilli*, in the hindgut, which leads to increased nitrogen uptake and decreased ammonia excretion [6,18]. Improving the utilization and absorption of nutrients, healthy modulation of the intestinal microbiota ecosystem, and lowering the pH of the litter cause reducing of contaminants excreted. The fact is of great interest to poultry farming because it shows an additional way to reduce the toxic impact on the environment [29,30]. The increase in the amount of excreted litter in the experimental groups is probably because lactulose applied separately or in combination with biologically active synergists improves the chicken’s appetite increases the digestibility of nutrients of the consumed feed, optimizes the digestive processes and has a laxative effect.

**Table 9.** Concentration of noxious gases emitted by litter of chickens aged 17 wk, ppm (mean ± S.E.M.).

Indicators	Group		
	CON (*n =* 24)	T1 (*n =* 24)	T2 (*n =* 24)
NH_3_	50.9 ± 2.7	39.5 ± 2.1^b^	38.2 ± 1.9^a^
H_2_S	7.5 ± 0.2	6.7 ± 0.2^b^	6.3 ± 0.1^a^
R-SH	6.2 ± 0.3	5.4 ± 0.2^c^	5.1 ± 0.2^b^

Note: a = *p* < 0.001; b = *p* < 0.01; and c = *p* < 0.05 compared with data on control group.

## Conclusion

Taken together, the scientific validity and practical feasibility of new lactulose-containing supplements in the poultry industry were studied on a replacement chicken flock of the egg-laying Hisex Brown cross and made it possible to provide a scientific justification for antibiotic replacement being effective for improving production indicators and health of the chickens, as well as to identify a new way to reduce the burden on the environment.

## List of Abbreviations

BW, Body Weight; CF, Crude Fiber; GI, Gastrointestinal; wk, week.
